# Knowledge and preventive practices regarding COVID-19 disease among Ukrainian refugees in Poland

**DOI:** 10.1097/MD.0000000000037833

**Published:** 2024-04-19

**Authors:** Ewa Sobieraj, Jakub Goławski, Anna Sikora, Łukasz Duda-Duma, Marcin Korzeń, Oskar Pasek, Klaudia Pyzio, Maria Gańczak

**Affiliations:** a Student Research Group, Collegium Medicum, University of Zielona Gora, Zielona Gora, Poland; b Department of Methods of Artificial Intelligence and Applied Mathematics, West Pomeranian University of Technology, Szczecin, Poland; c Department of Infectious Diseases, Collegium Medicum, University of Zielona Gora, Zielona Gora, Poland.

**Keywords:** attitudes, COVID19, knowledge, refugees, Ukraine, vaccination

## Abstract

Russia’s invasion of Ukraine contributed one of the largest migration movements in the 21st century. Refugees may become a source of severe acute respiratory syndrome coronavirus 2 infections for the residents of host countries. The study aim was to assess knowledge and preventive practices regarding coronavirus disease 2019 (COVID-19) among Ukrainian refugees in Poland. The cross-sectional study was conducted between March and April 2022 among Ukrainian refugees registering consecutively in Zielona Góra, Poland. Knowledge and preventive practices were assessed by giving 1 point for each correct answer by anonymous, self-administered questionnaire. The response rate was 96%, 190 participated (mean age 37.8 ± 15.5 years; 57.9% females); 61.6% self-reported their socio-economic status (SES) as high, 38.9% reported high level of education. The mean COVID-19 knowledge score was 3.06 ± 1.95; 19.5% scored >50%. The knowledge level was higher among migrants with high SES (*P* = .003). The mean preventive practices score was 2.56 ± 1.38; 54.0% scored ≥ 60%. 40.5% declared social distancing, 62.6% followed coughing etiquette, 69.0% home isolate themselves during COVID-19. 57.9% always used masks in public space, however 74.2% wore masks with uncovered nose. Refugees with higher education, high SES and knowledge level had significantly greater preventive practices scores (*P* = .002; *P* = .02; *P* = .03, respectively). The knowledge and preventive practices level was insufficient. Educational campaigns oriented to raising knowledge and prevention behavior skills should be implemented, especially targeting high-risk groups to avoid spread of COVID-19.

## 1. Introduction

Before the Russian’s invasion of Ukraine, 1.35 million migrants lived in Poland.^[1]^ The Russian aggression in February 2022 caused enormous exodus of Ukrainians to neighboring countries, including Poland.^[1]^ According to the latest report from August 2023, the Polish-Ukrainian border was crossed by 14.48 million war refugees,^[2]^ 96% of them were females. Of note, almost a half (48%) declared Poland as the final destination country.^[3]^ However, general mobilization into the Armed Forces and changes in the extent of war zone had caused migration’s homecoming. Therefore, currently, it is difficult to estimate the Ukrainian’s population size in Poland. Concerning the previous data, Ukrainian migrants are a mobile community with low immunization rates.^[4,5]^ For instance, less than 40% of Ukrainians have received the coronavirus disease 2019 (COVID-19) vaccine in 2022.^[5]^ This was an important factor which influenced epidemiological situation in the country: until March 7, 2023, Ukraine has reported 5.39 million severe acute respiratory syndrome coronavirus 2 (SARS-CoV-2) infections and nearly 111,300 deaths.^[6]^ Due to the large migration wave and relatively high vaccination hesitancy among refugees, there is difficult to provide efficient prevention regarding COVID-19 in the hosting countries.^[7]^ Refugees may become a potential source of infection for residents.^[8]^

Ukrainian refugees (UR) who came to Poland were located in the numerous refugee centers. Usually they stayed in large clusters, which may contribute to spread of infectious diseases, including air-borne infections. Only 34.6% of the Ukrainian population received complete primary series.^[9]^ This is a poor result compared to the immunization rate in the European Union, which is 83.4%.^[7]^ From July 2021 vaccinations in Ukraine also covered children and adolescents aged 12 to 18. In this age group, only 193,380 vaccinations with Comirnaty vaccine were performed.^[10]^ The low vaccination coverage among Ukrainian citizens is mainly due to serious concerns about the side effects of the vaccine and its poor quality.^[11]^

The latest report showed that refugees can use false vaccination certificates; this may have a significant impact on public health.^[11]^ Current research shows that following nonspecific methods of SARS-CoV-2 infection prevention may decrease the number of COVID-19 cases^[12]^ adequate knowledge may also have a significant impact on limiting the spread of infection.^[13]^

Not much research on attitudes and knowledge regarding this issue has been carried out among UR to date, therefore we decided to conduct that cross-sectional study. The study aim was to assess knowledge and preventive practices regarding COVID-19 among UR in Poland.

## 2. Materials and methods

### 2.1. Study design and setting

A cross-sectional study was conducted in March and April 2022 in the Zielona Gora City Hall Civil Affairs Department which is oriented to handling cases related to the legalization of stay of foreigners in Zielona Gora, Lubuskie province, Poland. Although it was available for all foreigners, at the time the study was conducted the vast majority of migrants were UR.

### 2.2. Study population

The study population consisted of UR, who came to Poland after the Russian aggression to Ukraine. Consecutive Ukrainians who registered in a refugee center were invited to participate in the study. Respondents were informed that participation was anonymous, voluntary and that they had full rights to withdraw from the study at any time. Inclusion criteria were as follows:

• being a UR
• signing an informed consent to participate.

### 2.3. Study instrument and data collection

Seven students of the medical faculty of the University of Zielona Góra, Poland, members of the Students’ Research Group, were handling out questionnaires to Ukrainians. A self-administered anonymous questionnaire was used as the data collection instrument. It was developed after thorough literature review.^[14–17]^ Filling out the questionnaire took about 5 to 10 minutes. The questionnaire consisted of 21 questions divided into 3 parts: demographic data (age, gender, residency, education, socio-economic status [SES], and marital status); knowledge about COVID-19; and attitudes toward COVID-19. We divided education into higher education level and lower education level. Higher education level means that participates graduated university. Knowledge about COVID-19 was assessed giving 1 point for each correct answer to 9 questions (a scale of 0–9). A total knowledge score was then calculated for each respondent. The higher the score, the better knowledge of COVID-19. Scores of 0 to 5 (≤55% of correct answers) were arbitrary taken as poor, 6 to 9 (more than 55% of correct answers) as adequate knowledge. Attitudes toward COVID-19 were assessed by giving 1 point for each correct answer to the 5 items regarding: following coughing etiquette; using masks in public spaces, wearing mask with covered nose, keeping social distance and practicing home isolation while experiencing SARS-CoV-2 infection syndromes. The scale measured attitudes from a minimum of 0 to a maximum of 5. Scores for individuals were summed up to give a total score. Scores of ≥3 points (≥60%) were taken as a adequate attitude level.

According to the rapport refugees population may have been around 575,100.^[18]^

The representative target sample size was calculated with a sample size calculator.^[19]^ This arrived at 384 participants, using a margin of error of ±5%, a confidence level of 95% and a 50% response distribution.

Anonymous cross-sectional surveys, like this one, do not require the consent of the Bioethical Committee Collegium Medicum University of Zielona Góra in Poland.

According to the statement posted on the official website of the Collegium Medicum of the University of Zielona Gora “*A relevant bioethics committee consent is not required in the case of a survey involving the use of questionnaires used in accordance with their intended purpose; statistical analysis will be developed based on selected elements of the survey.”*^[20]^

### 2.4. Statistical analysis

Data were converted to a Microsoft Excel version 16.53 and analyzed using STATISTICA PL Version 13 (StatSoft Inc., 2017) and a statistical software package (R Foundation for Statistical Computing, Vienna, Austria).^[21]^ Categorical variables were presented as frequencies with percentages to describe UR characteristics, and continuous data were given as means. Preventive practices regarding COVID-19 was our main variable. Demographic characteristics were assessed with bivariate analysis as follows: age (years), gender, residency in Ukraine (city of up to 50,000/>50,000 citizens), marital status (married-cohabitant/other), education level (higher/lower education) and COVID-19 knowledge level (adequate/poor). The Shapiro–Wilk test showed that variables were not normally distributed (*P* < .005), so the non-parametric tests were used to assess the significance of the difference. The Fisher’s exact test were used for 2 group comparisons of categorical variables. For numerical variables, the Mann–Whitney test was used. A *P* value was statistically significant if <.05. To build a logistic regression model,^[22]^ the set of predictors was used with the help of the R MASS package.^[23]^ Final associations between predictors and the outcome adjusted for covariates were measured with the use of coefficients of a logistic regression model to evaluate any changes in the model. Regression results are presented as odds ratios (ORs).

## 3. Results

### 3.1. Sociodemographic characteristics

The sociodemographic characteristics of the participants are presented in Table 1. Initially, 198 refugees were invited, 190 agreed to take part (the response rate was 96.0%). The mean age was 37.8 ± 15.7 years (range: 15–83 years); females accounted for 57.9%. More than a half of respondents (52.1%) were living in cities ≥50,000 inhabitants and were unmarried (55.8%). Almost two thirds (61.6%) self-assessed their SES as high; more than one third (39.0%) had a higher education level.

**Table 1:** Demographic characteristics of the studied population; Zielona Gora, Poland, 2022 (n = 190).

Variable		n	%
Age range: 15–83; mean 37.8 ± 15.5			
Gender	Female	110	57.9
	Male	80	42.1
Residency	≥50,000	99	52.1
	<50,000	91	47.9
Education	Higher	74	38.9
	Other	116	61.1
Self-assessed SES	High	117	61.6
	Low	73	38.4
Marital status	Married	84	44.2
	Other	106	55.8

SES = socio-economic status.

### 3.2. Knowledge on COVID-19

Participants were asked to self-assess their knowledge level about SARS-CoV-2/COVID-19; the answers were as follows: poor – 11.1% (21/190), adequate – 32.6% (62/190), very good – 32.6% (62/190), and “I don’t know” – 23.7% (45/190).

The mean knowledge score was 3.06 ± 1.95; 20 of 190 who responded to knowledge questions (10.5%) scored 0 points, 25 (13.2%)—scored 1 point, 28 (14.7%) scored 2 points, 40 (21.1%) scored 3 and 4 points, respectively, 18 (9.5%)—scored 5 points, 10 (5.3%) scored 6 points, 4 (2.1%) scored 7 points, 3 (1.6%) scored 8 points and 2 (1.1%) scored 9 points. Almost one-fifth (19.5%) of the migrants scored more than 50% of the correct answers. Only 10% (19/190) had adequate knowledge level.

UR knowledge level is presented in Table 2. About two-fifths knew the most effective type of mask, 54.7% indicated that COVID-19 is highly infectious. Less than 10% correctly recognized mode of transmission, 35.8% didn’t know that some infected persons may be asymptomatic. Less than half of respondents (41.6%) correctly marked COVID-19 incubation period, 26.3% knew that SARS-CoV-2 doesn’t leave lifelong immunity. About a quarter correctly recognized a higher COVID-19 mortality rate in older group then in younger people, 44.2% knew about an accessible drug reducing the risk of severe COVID-19. Only one-third (31.6%) knew that SARS-CoV-2 has animal origin.

**Table 2:** Ukrainian refugees knowledge about COVID-19; Zielona Gora, Poland, 2023 (n = 190).

Statement	Correct answer	True	
		*n*	%
The most effective type of mask	Mask with FFP2/3 filter	70	36.8
COVID-19 is a highly infectious disease	Yes	104	54.7
Transmission of COVID-19	Droplet, surface transmission	18	9.5
An infected person always shows symptoms of the disease	No	68	35.8
The incubation period of COVID-19	2–14 d	79	41.6
SARS-CoV-2 infection leaves lifelong immunity	No	50	26.3
COVID-19 mortality rate at age 65–75 is higher than at age 18–30	10 times	47	24.7
Accessible drug to reduce risk of severe COVID-19	Yes	84	44.2
SARS-CoV-2 has animal orgin	Yes	60	31.6

COVID-19 = coronavirus disease 2019, SARS-CoV-2 = severe acute respiratory syndrome coronavirus 2.

Table 3 presents knowledge on COVID-19 among UR in Poland by selected variables. Participants with higher SES demonstrated greater knowledge (3.14 ± 1.94) than respondents with lower SES (2.92 ± 1.99); *P* = .003. There were no statistically significant differences between UR regarding age, gender, education, marital status, and residence in Ukraine.

**Table 3:** Knowledge on COVID-19 among Ukrainian refugees by selected variables, Zielona Gora, Poland, 2022 (n = 190).

Variable		Correct answer	Mean	SD	*P* value
Age (yr)	<38	36.8%	3.31	2.12	.22
	≥38	30.4%	2.73	1.68	
Gender	Male	27.8%	2.50	1.67	.91
	Female	38.5%	3.46	2.05	
Residence in Ukraine (city)	<50,000 residents	31.3%	2.81	1.84	.15
	≥50,000 residents	36.4%	3.27	2.02	
Marital status	Married	32.0%	2.88	1.79	.82
	Other marital status	35.5%	3.20	2.07	
Self-reported SES	High SES	34.9%	3.14	1.94	.003
	Low SES	32.4%	2.92	1.99	
Education	Higher education	35.9%	3.23	2.05	.08
	Other education	32.8%	2.95	1.89	

COVID-19 = coronavirus disease 2019, SES = socio-economic status.

### 3.3. Preventive practices

More than a half of UR (57.9%) used masks in the public space, however, about three-fourth of participants (74.2%) declared then they wore masks with uncovered nose. Almost two in three (62.6%) respondents followed coughing etiquette, only 40.5% kept social distancing. More than three-thirds (69.0%) were practicing home isolation while experiencing SARS-CoV-2 infection syndromes.

The mean practices score was 2.56 ± 1.38. Only 53.7% of respondents scored ≥60% of correct answers. Preventive practices score was significantly higher in the group with higher education level (2.95 ± 1.34) than with lower education level (2.31 ± 1.35) and with high SES (2.74 ± 1.35) than with low SES (2.26 ± 1.39); *P* = .002 and *P* = .02, respectively. There were no statistically significant differences between attitudes scores in terms of age, gender, residence in Ukraine and marital status (Table 4). UR with higher COVID-19 knowledge level scored more regarding the preventive practices score (3.21 ± 0.98 vs 2.49 ± 1.40); *P* = .03.

**Table 4:** Preventive practices towards COVID-19 among Ukrainian refugees by selected variables, Zielona Gora, Poland, 2022 (n = 190).

Variable		Adequate practice	Mean	SD	*P* value
Age	<38	51.0%	2.55	1.45	.88
	≥38	50.9%	2.54	1.30	
Gender	Male	51.0%	2.55	1.29	.95
	Female	51.3%	2.56	1.45	
Residence in Ukraine (city)	<50,000 residents	48.1%	2.41	1.41	.15
	≥50,000 residents	53.9%	2.70	1.35	
Marital status	Married	54.3%	2.71	1.32	.16
	Other marital status	48.7%	2.43	1.42	
Self-reported SES	High SES	54.9%	2.74	1.35	.02
	Low SES	45.2%	2.26	1.39	
Education	Higher education	58.9%	2.95	1.34	.002
	Other education	46.2%	2.31	1.35	
Knowledge level	High	64.2%	3.21	0.98	.03
	Low	49.7%	2.49	1.40	

COVID-19 = coronavirus disease 2019, SES = socio-economic status.

### 3.4. Predictors of being adherent to preventive practices

Multiple logistic regression analysis regarding an association of being adherent to COVID-19 preventive practices with selected variables revealed that education status and knowledge level were independent positive predictors of presenting adequate practices (OR 2.19; *P* = .03 and OR 4.01; *P* < .0001, respectively); Table 5.

**Table 5:** Logistic regression model: association of COVID-19 preventive practices with selected variables: estimates and *P* values (n = 190).

Variable	Estimate	*P*
Intercept	3.10	<.001
Gender (male)	0.05	.78
Residence in Ukraine (<50,000 residents)	0.22	.83
Marital status (other)	0.28	.14
Self-reported SES (low)	0.04	.60
Educational status (high)	2.19	.03*
Knowledge level (high)	4.01	<.0001*

COVID-19 = coronavirus disease 2019, SES = socio-economic status.

* Statistically significant.

## 4. Discussion

### 4.1. Result overview

To the best of our knowledge, this was the first study about knowledge and preventive practices towards COVID-16 among UR in Poland. Previous studies among Ukrainian migrants were focused on vaccinations.^[1,4]^ Our findings show that UR presented poor knowledge level about COVID-19 and declared inadequate attitudes regarding prevention practices, particularly those with lower education (OR = 2.2) and knowledge level (OR = 4.0), suggesting that well educated subjects with high knowledge would practice more preventive measures.

### 4.2. Knowledge towards COVID-19

According to the previous research, knowledge affects the formation of attitudes and practices.^[24]^ Checking the knowledge level in migrants is important to detect gaps, which can be further fulfilled through tailored education campaigns.

Only one in five refugees answered correctly to at least half of the questions. The knowledge level was lower than reported in other recently published studies.^[24–27]^ However, these studies were conducted on different group of participants (not targeting migrants) and used online questionnaires; this means that external sources of information could have been used.

Less than 1 in 10 migrants knew the mode of transmission COVID-19, which is alarming due to the fact that low knowledge can result in non-adherence to COVID-19 prevention methods. There is a risk that people with poor knowledge of transmission pathways could become a potential source of infection.^[28]^

Fewer than 20% knew that surviving COVID-19 does not provide lifelong immunity. We believe that such reasoning can negatively affect the use of methods to prevent the future episodes of the disease once it has been contracted. However, further research is needed.

Migrants distinguished themselves with the highest knowledge regarding the fact that COVID-19 is a highly contagious disease. This information often appeared in the media which may have influenced on the number of correct answers. However, almost half of the respondents chose the wrong answer, which could affect the spread of COVID-19. Notably, this message was identified in fake news appearing in social media since the beginning of the pandemic.^[29]^

Data from the literature described an association between knowledge, high-income and education.^[30]^ We have shown that knowledge level about COVID-19 was specifically low in those with self-reported poor SES. In general, individuals of lower SES have less health-related knowledge. Supporting this idea, research early in the COVID-19 epidemic found lower COVID-related knowledge among individuals of lower SES.^[31,32]^

### 4.3. Preventive practices towards COVID-19

We have shown that COVID-19 related preventive practices were inappropriate, particularly mask usage with uncovered nose. Appropriate masks usage is crucial to reduce the likelihood of SARS-CoV-2 infection due to the interruption of the virus transmission path.^[33,34]^

Problems with maintaining appropriate practices in Ukraine often result from the lack of availability of personal protective equipment. At the beginning of the pandemic, masks were unavailable and then expensive.^[35]^ This forced the population to use masks many times, which did not ensure adequate effectiveness. Part of seniors groups complained that masks are made a way which make them difficult to breathe; therefore they refrained from using them.^[36]^

A little more than a half of our respondents declared using masks in public spaces. This is less than reported in other published studies. For instance, research conducted in Poland showed that about 60.4% of participants declared the use of masks in public space.^[37]^ Another observational study showed that the percentage of those using masks was 73.6% at the first time point, decreasing after a week to 66.5% and 2 weeks later to 65.7%.^[38]^ However, when the study was conducted there were not mandatory masks wearing in public space. That fact may have contributed to the deterioration of practices. Residents of Romania and Bosnia and Herzegovina showed cautious practices with as much as 95.7% and 91.9% of subjects respectively wearing a face mask while being outside of their home.^[39,40]^ However, a survey conducted in 38 countries on 13,723,810 participants shows that some countries such as Chile, Italy and Japan had consistently high mask usage (>75%) compared to the countries with low usage mask (<25%) (Denmark, Sweden, and Norway).^[41]^

Only two-thirds of our respondents declared following coughing etiquette. Previous research suggests better adherence to coughing etiquette among refugees^.[42]^ Four years of observing coughing etiquette of people in public locations, in the United States, resulted in observing less than 2 percent properly covering their nose and mouth while coughing or sneezing. Of note, that study was conducted before the COVID-19 pandemic.^[43]^ Further research on following coughing etiquette is urgently needed.

The present study shows that a large majority of UR declared wearing mask with uncovered nose. This was in line with other study conducted in Japan that showed only 40.8% of participants placing the mask ensuring it covers the mouth and nose.^[44]^ This may be due to the fact that masks are perceived as uncomfortable.^[45]^ Masks usage in Japan have been introduced as a response to the Spanish flu in 1919. Since then, wearing a face mask has been entrenched as a protection against pollution and used as a general self-protective risk ritual.^[46]^ Before the pandemic, there were no widely available recommendations about wearing masks in most of the countries.

Only 40.5% of UR declared keeping social distance. The results are in line with those reported in a study conducted in the United States which showed that only about one third of participants reported social distancing in May 2020.^[47]^ However, most recent studies suggest more effective maintaining of social distancing; it was at a higher level than declared among UR.^[42,48,49]^

The vast majority of our respondents declared that they stayed in home isolation during COVID-19 infection. This was concordant with the results of one previous survey published in the United Kingdom.^[50]^ Better home isolation was reported among residents of Iran.^[39]^

The better education level and higher knowledge scores was significantly associated with better practices scores, meaning that more educated participants with a high level of knowledge were engaged in more prevention practices. This might be due to the fact that these groups are more aware of health-seeking behaviors, better understand the preventive measures, the rationales for their use and their impact on health.^[40]^ Similar results were reported by Šljivo et al;^[51,52]^ subjects who had higher knowledge test score were more prone to respect socio-epidemiological measures, such as wearing a mask while outside the home. Other authors have also previously reported associations when performing knowledge, attitudes and practices surveys in some other infectious diseases.^[53]^ Better knowledge may result in good practices, thus aiding in the prevention and management of infectious diseases. Our study may help to set national public health campaigns priorities in order to address the most misunderstood and hazardous practices.

## 5. Limitations

A key limitation of the current study was the small sample size which may influence the generalizability. This was due to the small number of refugees who registered in the refugee center in the dedicated time period. Additionally, the convenience sampling is another shortcoming of our survey. Convenient sampling has disadvantages related to population bias that may limit the extrapolation of the results in the target population. The study employed a convenient sample since time is of essence in COVID-19 research and thus the sample may not be representative of all UR in Poland. Nevertheless, we believe that the data presented here could be considered as a satisfactory reflection of the knowledge, and practices of UR regarding SARS-CoV-2 infection prevention, given that our sample included migrants which differed regarding age, gender, residency in Ukraine, marital status and education level. Second, data was collected only in one refugee center. Further studies should be conducted in other parts of the country, varying in terms of population size and region. Third, questionnaire was distributed in Ukrainian language. Therefore, UR may not fully understand the questions. However, if a question was difficult to understand, respondents could get help from research team member present on the site. Fourth, while we highlighted some variables regarding the UM demographic, other factors, for example, religion, as well as masks availability and affordability might have also influenced risky practices. Finally, we did not make an objective assessment of practices. Migrants could make answers, which they thought as the most desirable.

## 6. Conclusions

We found that knowledge and practices regarding nonspecific methods of preventing SARS-CoV-2 infection among UM in Poland were unsatisfactory. Efforts should be made to improve knowledge and practices in this group to better control the pandemic. Health education programs should be implemented on the national and regional level to effectively raise the COVID-19 knowledge level and better equip UR with COVID-19 prevention skills. Health education programs should be prepared in a language understood by refugees, in particular among those with lower education and lower SES.

## Acknowledgments

Authors would like to thank Ukrainian refugees who enthusiastically participated in the study. Special thanks to Prof Serhij Nyankowskyy, Collegium Medicum, University of Zielona Gora, for his valuable help with literature review.

## Author contributions

**Conceptualization:** Ewa Sobieraj.

**Data curation:** Ewa Sobieraj, Jakub Goławski, Anna Sikora, Łukasz Duda-Duma, Marcin Korzeń, Oskar Pasek, Klaudia Pyzio.

**Formal analysis:** Ewa Sobieraj, Jakub Goławski, Marcin Korzeń, Maria Gańczak.

**Investigation:** Ewa Sobieraj, Jakub Goławski, Anna Sikora, Łukasz Duda-Duma, Oskar Pasek, Klaudia Pyzio.

**Resources:** Ewa Sobieraj, Jakub Goławski, Anna Sikora, Maria Gańczak.

**Supervision:** Maria Gańczak.

**Writing – original draft:** Ewa Sobieraj, Jakub Goławski, Maria Gańczak.

**Writing – review & editing:** Ewa Sobieraj, Maria Gańczak.

## References

[1] Central Statistical Office of Poland. *Population migration report*. stat.gov.pl. Available at: https://stat.gov.pl/obszary-tematyczne/ludnosc/migracje-zagraniczne-ludnosci. Accessed March 23, 2024.

[2] Malchrzak W Babicki M Pokorna-Kałwak D. Covid-19 vaccination and Ukrainian refugees in Poland during Russian–Ukrainian War–narrative review. Vaccines. 2022;10:955. 35746562 10.3390/vaccines10060955 PMC9230022

[3] *Official profile of the Polish Border Guard on the social network Twitter*. Twitter. Available at: https://twitter.com/i/flow/login?redirect_after_login=%2FStraz_Graniczna. Accessed March 23, 2024.

[4] Rzymski P Falfushynska H Fal A. Vaccination of Ukrainian refugees: need for urgent action. Clin Infect Dis. 2022;75:1103–8. 35435230 10.1093/cid/ciac276 PMC9383728

[5] National Vaccination Schedule Approved by Ministry of Health of Ukraine in 2022. Ministry of Health of Ukraine. Available at: https://www.phc.org.ua/kontrol-zakhvoryuvan/imunizaciya/zagalna-informaciya. Accessed March 23, 2024.

[6] Korrespondent.net. *В Украине сделано более 31 млн covid-прививок*. Новости Украины и мира. Available at: https://korrespondent.net/ukraine/4450254-v-ukrayne-sdelano-bolee-31-mln-COVID-pryvyvok. Accessed March 23, 2024.

[7] World Health Organization. Covid-19 Cases | WHO COVID-19 Dashboard. World Health Organization; n.d. Available at: https://covid19.who.int/region/euro/country/ua. Accessed April 01, 2023.

[8] Sirkeci I Yucesahin MM. Coronavirus and migration: analysis of human mobility and the spread of covid-19. Migr Lett. 2020;17:379–98.

[9] Apart Together Survey, Preliminary Overview of Refugees and Migrants Self-reported Impact of Covid-19 - World. ReliefWeb; 2020. Available at: https://reliefweb.int/report/world/aparttogether-survey-preliminary-overview-refugees-and-migrants-self-reported-impact. Accessed March 23, 2024.

[10] Safe Covid-19 Vaccines for Europeans. European Commission; n.d. Available at: https://commission.europa.eu/strategy-and-policy/coronavirus-response/safe-covid-19-vaccines-europeans_en. Accessed November 20, 2023.

[11] National Institute of Public Health - National Institute of Hygiene. Co Wiemy na temat Stanu Zaszczepienia Dzieci W Ukrainie Przed Chorobami Zakaźnymi przed KTÓRYMI Chronią Szczepienia? Szczepienia.Info.; 2022. Available at: https://szczepienia.pzh.gov.pl/faq/co-wiemy-na-temat-stanu-zaszczepienia-dzieci-w-ukrainie-przed-chorobami-zakaznymi-przed-ktorymi-chronia-szczepienia. Accessed June 15, 2023.

[12] Ganczak M Kalinowski P Pasek O. Health system barriers to child mandatory and optional vaccination among Ukrainian migrants in Poland in the context of MMR and HPV vaccines – a qualitative study. Int J Environ Res Public Health. 2022;20:712. 36613034 10.3390/ijerph20010712 PMC9819946

[13] Rahman S Montero MT Rowe K. Epidemiology, pathogenesis, clinical presentations, diagnosis and treatment of COVID-19: a review of current evidence. Expert Rev Clin Pharmacol. 2021;14:601–21. 33705239 10.1080/17512433.2021.1902303 PMC8095162

[14] Zhang M Zhou M Tang F. Knowledge, attitude, and practice regarding covid-19 among healthcare workers in Henan, China. J Hosp Infect. 2020;105:183–7. 32278701 10.1016/j.jhin.2020.04.012 PMC7194961

[15] Popa AD Antoniu SA Enache AI. Development and validation of a questionnaire to assess knowledge and attitudes toward covid-19 preventive measures in Romania. Healthcare (Basel). 2022;10:1827. 36292275 10.3390/healthcare10101827 PMC9601972

[16] Zaprutko T Kremin Y Michalak M. Social attitude to covid-19 and influenza vaccinations after the influenza vaccination season and between the second and third covid-19 wave in Poland, Lithuania, and Ukraine. Int J Environ Res Public Health. 2022;19:2042. 35206232 10.3390/ijerph19042042 PMC8871771

[17] Duszczyk M Kaczmarczyk P. Wojna i migracja: napływ uchodźców wojennych z Ukrainy i możliwe scenariusze na przyszłość. CMR Spotlight. 2022;4. Available at: https://www.migracje.uw.edu.pl/wp-content/uploads/2022/04/Spotlight-APRIL-2022-PL.pdf. Accessed June 23, 2023.

[18] Wojna Rosji przeciw Ukrainie – Stan po tygodniu. OSW Ośrodek Studiów Wschodnich; 2022. Available at: https://www.osw.waw.pl/pl/publikacje/analizy/2022-03-03/wojna-rosji-przeciw-ukrainie-stan-po-tygodniu. Accessed April 21, 2023.

[19] Sample Size Calculator by Raosoft, Inc. Web survey software, online surveys, email surveys from Raosoft, Inc. n.d. Available at: http://www.raosoft.com/samplesize.html. Accessed March 18, 2023.

[20] University of Zielona Góra. Bioethics Committee. bioethics committee University of Zielona Góra; n.d. Available at: https://cm.uz.zgora.pl/badania/komisja-bioetyczna/badania-naukowe-niesponsorowane. Accessed March 01, 2022.

[21] R Development Core Team. A Language and Environment for Statistical Computing. Vienna, Austria: R Foundation for Statistical Computing. Mining the Most Interesting Rules. Available at: http://www.R-project.org. Accessed November 22, 2023.

[22] Hosmer D Lemeshow S. Applied Logistic Regression. 2nd ed. New York, NY; Chichester, UK; Weinheim, Germany; Brisbane, Australia; Singapore; Toronto, ON, Canada: John Wiley & Sons Inc; 2000:95. Available at: https://ftp.idu.ac.id/wp-content/uploads/ebook/ip/REGRESI%20LOGISTIK/epdf.pub_applied-logistic-regression-wiley-series-in-probab.pdf. Accessed March 01, 2022.

[23] Venables WN Ripley BD. Modern Applied Statistics with S. 4th ed. New York, NY: Springer. 2002.

[24] Puspitasari IM Yusuf L Sinuraya RK. Knowledge, attitude, and practice during the COVID-19 pandemic: a review. J Multidiscip Healthc. 2020;13:727–33. 32801735 10.2147/JMDH.S265527 PMC7407756

[25] Masoud AT Zaazouee MS Elsayed SM.; KAP-COVIDGLOBAL Investigators. Kap-Covid global: a multinational survey of the levels and determinants of public knowledge, attitudes and practices towards covid-19. BMJ Open. 2021;11:e043971. 10.1136/bmjopen-2020-043971 PMC7907623 33622949

[26] Lee M Kang BA You M. Knowledge, attitudes, and practices (KAP) toward covid-19: a cross-sectional study in South Korea. BMC Public Health. 2021;21:295. 33546644 10.1186/s12889-021-10285-y PMC7863060

[27] Šljivo A Abdulkhaliq A Granov N. COVID-19 vaccination knowledge, attitudes and practices among the general population of Romania during the third wave of COVID-19 pandemic. SAGE Open Med. 2023;11:20503121231165670. 37089469 10.1177/20503121231165670 PMC10111160

[28] Washif JA Farooq A Krug I. Training during the COVID-19 lockdown: knowledge, beliefs, and practices of 12,526 athletes from 142 countries and six continents. Sports Med. 2021;52:933–48. 34687439 10.1007/s40279-021-01573-z PMC8536915

[29] Yanti B Wahyudi E Wahiduddin W. Community knowledge, attitudes, and behavior towards social distancing policy as prevention transmission of COVID-19 in Indonesia. J Administrasi Kesehatan Indonesia. 2020;8:4.

[30] Šljivo A Bhattacharyya S Mulać A. Knowledge, attitudes and practices during the second wave of COVID-19 outbreak: a cross-sectional study from various perspectives. Med Glas (Zenica). 2021;18:499–504. 10.17392/1378-21 34212710

[31] Ostrovsky AM Chen JR. TikTok and its role in covid-19 information propagation. J Adolesc Health. 2020;67:730. 32873499 10.1016/j.jadohealth.2020.07.039 PMC7455791

[32] Siddiquea BN Shetty A Bhattacharya O. Global epidemiology of COVID-19 knowledge, attitude and practice: a systematic review and meta-analysis. BMJ Open. 2021;11:e051447. 10.1136/bmjopen-2021-051447 PMC8441223 34521674

[33] Liao M Liu H Xi W. A technical review of face mask wearing in preventing respiratory COVID-19 transmission. Curr Opin Colloid Interface Sci. 2021;52:101417. 33642918 10.1016/j.cocis.2021.101417 PMC7902177

[34] Chen W. Multi-agent economic analysis in coping with novel corona-virus pneumonia. J Phys Conf Ser. 2021;1812:012018.

[35] Філатов В. *Пропали з продажу і подорожчали. Чому медичні маски у Вінниці стали дефіцитом - 20 хвилин*. n.d. Available at: https://vn.20minut.ua/Zdorovya/propali-z-prodazhu-i-podorozhchali-chomu-medichni-maski-u-vinnitsi-sta-11038025.html. Accessed June 26, 2023.

[36] Маска на подборідді не рятує від коронавірусу: чому кияни нехтують правилами безпе. n.d. Available at: https://vechirniy.kyiv.ua/news/46736. Accessed June 29, 2023.

[37] Matusiak Ł Szepietowska M Krajewski PK. The use of face masks during the COVID-19 pandemic in Poland: a survey study of 2315 young adults. Dermatol Ther. 2020;33:e13909. 32602208 10.1111/dth.13909 PMC7361243

[38] Ganczak M Pasek O Duda-Duma Ł Świstara D Korzeń M. Use of masks in public places in Poland during SARS-CoV-2 epidemic: a covert observational study. BMC Public Health. 2021;21:393. 33622279 10.1186/s12889-021-10418-3 PMC7901005

[39] Shahabi N Takhti HK Azad MH. Knowledge, attitude, and preventive behaviors of Hormozgan residents toward COVID-19, one month after the epidemic in Iran. J Public Health. 2021;30:1565–76. 33432291 10.1007/s10389-020-01454-1 PMC7788169

[40] Wang C Tian Q Zhao P. Disease knowledge and attitudes during the COVID-19 epidemic among international migrants in China: a national cross-sectional study. Int J Biol Sci. 2020;16:2895–905. 33061804 10.7150/ijbs.47075 PMC7545702

[41] Badillo-Goicoechea E Chang T Kim E. Global trends and predictors of face mask usage during the COVID-19 pandemic. BMC Public Health. 2021;21:2099. 34781917 10.1186/s12889-021-12175-9 PMC8667772

[42] Van Nhu H Tuyet-Hanh TT Van NTA. Knowledge, attitudes, and practices of the Vietnamese as key factors in controlling COVID-19. J Community Health. 2020;45:1263–9. 32894387 10.1007/s10900-020-00919-4 PMC7476242

[43] Wolff RJ. No cover-up here: a descriptive study of observations of coughing on hands and the lack of proper respiratory hygiene behaviors or cough etiquette. Soc Sci Res Netw. 2020.

[44] Machida M Nakamura I Saito R. Incorrect use of face masks during the current COVID-19 pandemic among the general public in Japan. Int J Environ Res Public Health. 2020;17:6484. 32899922 10.3390/ijerph17186484 PMC7557398

[45] Scheid JL Lupien SP Ford GS. Commentary: physiological and psychological impact of face mask usage during the COVID-19 pandemic. Int J Environ Res Public Health. 2020;17:6655. 32932652 10.3390/ijerph17186655 PMC7558090

[46] Burgess A Horii M. Risk, ritual and health responsibilisation: Japan’s ‘safety blanket’ of surgical face mask-wearing. Sociol Health Illn. 2012;34:1184–98. 22443378 10.1111/j.1467-9566.2012.01466.x

[47] Gharpure R Hunter CM Schnall AH. Knowledge and practices regarding safe household cleaning and disinfection for COVID-19 prevention – United States, May 2020. Morb Mortal Wkly Rep. 2020;69:705–9. 10.15585/mmwr.mm6923e2 PMC7315790 32525852

[48] Bates BR Moncayo AL Costales JA. Knowledge, attitudes, and practices towards COVID-19 among Ecuadorians during the outbreak: an online Cross-Sectional survey. J Community Health. 2020;45:1158–67. 32915380 10.1007/s10900-020-00916-7 PMC7483492

[49] Sulistyawati S Rokhmayanti R Aji B. Knowledge, attitudes, practices and information needs during the COVID-19 pandemic in Indonesia. Risk Manag Healthc Policy. 2021;14:163–75. 33488129 10.2147/RMHP.S288579 PMC7814231

[50] Smith L Amlôt R Lambert H. Factors associated with adherence to self-isolation and lockdown measures in the UK: a cross-sectional survey. Public Health. 2020;187:41–52. 32898760 10.1016/j.puhe.2020.07.024 PMC7474581

[51] Šljivo A Ćetković A Abdulkhaliq A. COVID-19 vaccination knowledge, attitudes and practices among residents of Bosnia and Herzegovina during the third wave of COVID-19 outbreak. Ann Ig. 2022;34:490–500. 34821929 10.7416/ai.2021.2489

[52] Šljivo A Kačamaković M Siručić I. Knowledge, attitudes, and practices towards COVID-19 among residents of Bosnia and Herzegovina during the first stage of COVID-19 outbreak. Ann Ig. 2021;33:371–80. 33908602 10.7416/ai.2021.2447

[53] Ul Haq N Hassali MA Shafie AA. A cross sectional assessment of knowledge, attitude and practice towards hepatitis B among healthy population of Quetta, Pakistan. BMC Public Health. 2012;12:692. 22917489 10.1186/1471-2458-12-692 PMC3490724

